# Sorbitol as a Chain Extender of Polyurethane Prepolymers to Prepare Self-Healable and Robust Polyhydroxyurethane Elastomers

**DOI:** 10.3390/molecules23102515

**Published:** 2018-09-30

**Authors:** Sang Hyub Lee, Se-Ra Shin, Dai-Soo Lee

**Affiliations:** Department of Semiconductor and Chemical Engineering, Chonbuk National University, 567 Baekje-daero, Deokjini-gu, Jeonju 54896, Korea; shlee87@jbnu.ac.kr (S.H.L.); sera2429@gmail.com (S.-R.S.)

**Keywords:** sorbitol, self-healing, polyhydroxyurethane, exchange reaction

## Abstract

A self-healable polyhydroxyurethane (S-PU) was synthesized from sorbitol, a biomass of polyhydric alcohol, by a simple process that is suitable for practical applications. In the synthesis, only two primary hydroxyl groups of sorbitol were considered for the chain extension of the polyurethane (PU) prepolymers to introduce free hydroxyl groups in PU. As a control, conventional PU was synthesized by hexane diol mediated chain extension. Relative to the control, S-PU showed excellent intrinsic self-healing property via exchange reaction, which was facilitated by the nucleophilic addition of the secondary hydroxyl groups without any catalytic assistance and improved tensile strength due to the enhanced hydrogen bonding. We also investigated the effect of the exchange reaction on the topological, mechanical, and rheological properties of S-PU. The suggested synthetic framework for S-PU is a promising alternative to the conventional poly hydroxyurethane, in which cyclic carbonates are frequently reacted with amines. As such, it is a facile and environmentally friendly material for use in coatings, adhesives, and elastomers.

## 1. Introduction

Polyurethane (PU) is one of the most versatile polymers. It is widely used in foams, coatings, adhesives, and elastomers due to its excellent mechanical properties and chemical resistance. Moreover, its properties are readily adjusted by varying either the process or the compositions of the isocyanates and polyols constituting its backbone via polymerization [1,2,3,4]. Its versatility has sparked great interest in self-healable PUs with higher durability when compared to the standard PUs. Moreover, self-healable materials are eco-friendly and cost-effective [5,6,7,8,9,10,11,12,13,14,15,16,17,18,19,20,21,22,23,24,25,26,27,28,29,30,31,32].

The development of self-healable PUs has significantly advanced in recent decades. The self-healability and reprocessability of PUs has been achieved by several strategies based on reversible dynamic covalent chemistry, such as the Diels–Alder (DA) and retro-DA reactions [11,12,13,14], disulfide metathesis [15,16,17,18,19,20,21,22,23], alkoxyamine chemistry [24], the amine–urea exchange reaction [25], and the reversible acetal chemistry [26]. However, dynamic chemistry brings into play the functional compounds into the PU system, thereby increasing the cost of the raw materials and the process complexity. Furthermore, the introduction of dynamic chemistry into the urethane system frequently not only aggravates the mechanical properties but the practical applicability of PUs synthesized by dynamic chemistry would also be restricted.

The transcarbamoylation reaction (TCR) is a dynamic chemistry based on exchange reactions among the carbamate groups. The TCR has recently attracted more attention because it achieves excellent self-healability and reprocessability of the PU network system based on the dynamic chemistry [27,28,29,30,31]. Unfortunately, the TCR of PUs generally requires high temperature (>200 °C) without catalysts, and decomposes or dissociates the carbamate linkages, resulting in adverse side reactions. Dichtel et al. synthesized a polyhydroxyurethane (PHU) vitrimer from a six-membered cyclic carbonate. They reported that the nucleophilic addition of a free hydroxyl group to the carbamate group enables the exchange reaction among the carbamate groups under relatively mild conditions, unlike the harsh conditions of previous reports [32]. However, the secondary reaction and low reactivity between the cyclic carbonate and amine derivatives were reported to lower the average molecular weight of cyclic carbonate-based PHUs [33,34,35,36], and the side reactions at high temperatures lowered the mechanical properties of PHUs [30,32]. Furthermore, manufacturing PHUs from cyclic carbonates is a slow, complex process than processing conventional PUs. Thus, it is difficult to upscale cyclic carbonate-based PHUs to industrial applications. Practical applicability of PHU preparation requires a simpler manufacturing process.

Sorbitol is a sugar alcohol obtained from the reduction of glucose [37,38,39]. This renewable resource is a common ingredient in food, cosmetics, medical applications, and polymer syntheses [40,41,42,43,44]. As the sorbitol molecule contains two primary hydroxyl groups and four secondary hydroxyl groups, it may be considered a chain extender of N=C=O-terminated PU prepolymers. Most primary –OH groups are expected to react with the N=C=O group of the prepolymer, thereby allowing the chain extension reaction, while most secondary –OH groups remain as free –OH units due to the large difference of reactivity toward the isocyanate groups [45,46,47]. Free –OH groups in PU may accelerate the activation of the TCR under mild conditions through nucleophilic addition to carbamate groups [32]. Inspired by these revelations, we simply prepared self-healable PHUs using sorbitol as the chain extender of conventional PU prepolymers. The prepared sorbitol-extended PU (S-PU) exhibited stronger mechanical properties and higher self-healing efficiency than the control PU chain extended with a short diol, 1,6-hexane diol. The short-term heating without any catalyst increased the excellent self-healing property of S-PU through the exchange reactions of the carbamate groups via nucleophilic addition of the hydroxyl groups. To the best of our knowledge, there was no report to use sorbitol as a chain extender of PU prepolymers, thereby generating PHU elastomeric systems with self-healing ability and enhanced mechanical properties.

## 2. Experiment

### 2.1. Materials

4,4′-Methylene-bis(phenyl isocyanate) (MDI) was purchased from Aldrich Chemical (Young-in) in Korea and used as received. Poly(tetramethylene ether glycol) (PTMEG) (Number average molecular weight (Mn): 2000 g/mol), sorbitol, and 1,6-hexane diol (HD), also purchased from Aldrich Chemical (Young-in) in Korea, were vacuum-dried at 60 °C for 1 day prior to use. Dimethylformamide (DMF) was used for solution casting of the prepared S-PU and H-PU.

### 2.2. Synthesis of PU Prepolymers

PU prepolymers were synthesized through the one-pot reaction between MDI and PTMEG without any solvent or catalyst. First, 2 mol of MDI and 1 mol of PTMEG were placed in a round-bottomed flask reactor with N_2_ gas purging at 50 °C and stirred to form a homogeneous mixture. After homogeneous mixing, the reaction temperature increased to 60 °C and the mixing was continued until the NCO% of the resultant reached its theoretical value. The NCO% change was determined following the ASTM D1638-74. Once the NCO% had reached its theoretical value, the reaction was completed and the prepared PU prepolymer was dissolved in DMF (at a weight ratio of prepolymer:DMF = 2:1).

### 2.3. Preparation of S-PU and H-PU

**Scheme 1:**
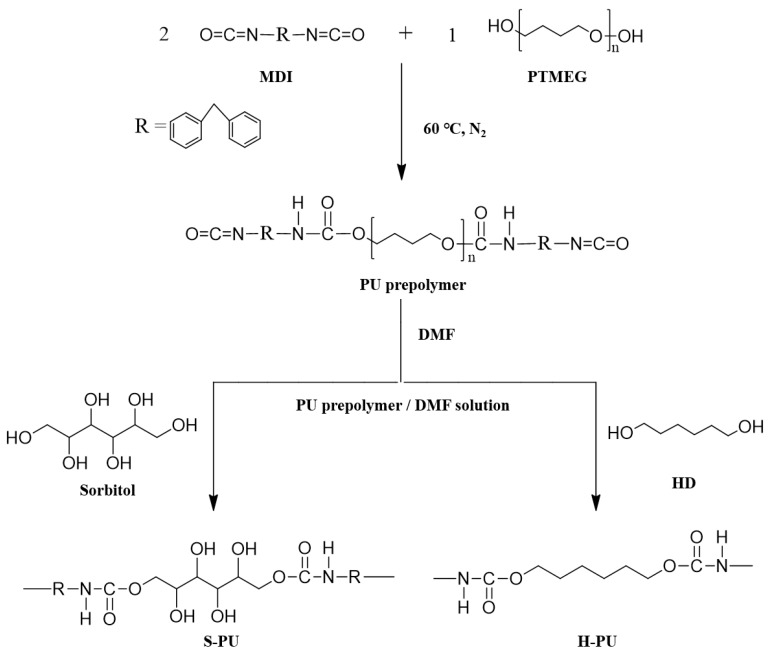
Preparation of S-PU and H-PU from isocyanate-terminated PU prepolymers.

The prepared PU prepolymer/DMF solution was mixed with 1,6-HD or sorbitol, initiating the chain extension of the N=C=O-terminated PU prepolymer. The stoichiometric amounts of HD and sorbitol were calculated based on the NCO% determined by ASTM D1638-74, sorbitol being considered diol. The PU prepolymer/DMF solution and chain extenders were thoroughly mixed by mechanical stirring for 1 h at room temperature, and then poured into a Teflon mold. The mold containing the homogenous mixture was placed in a 110 °C convection oven for 1 day to remove the DMF and to facilitate the polymerization of the PU prepolymer by its chain extenders. The PU films prepared by chain extension of sorbitol and HD were named S-PU and H-PU, respectively. The preparation steps and typical chemical structures of S-PU and H-PU are shown in Scheme 1.

### 2.4. Characterization

The chemical structures of the prepared samples were characterized by FT-IR spectroscopy (FT/IR 300E, JASCO, Easton, MD, USA). In addition, the exchange reaction of S-PU was investigated using a thermally controllable IR pellet loader. The potassium bromide (KBr) tablet was placed on the FT-IR sample loader and its temperature was successfully controlled by N_2_ gas purging. The molecular weights and polydispersity indices (PDIs) were determined by gel permeation chromatography (GPC) (Alliance e2695, Waters, Milford, MA, USA). The calibration curves for GPC were prepared using polystyrene standards. The GPC apparatus was equipped with a refractive index detector, and the eluent used was DMF flowing at 0.4 mL/min. Differential scanning calorimetry (DSC), (Q20, TA Instrument, New Castle, DE, USA) was carried out at a heating rate of 10 °C/min with N_2_ gas purging. The dynamic mechanical properties and stress relaxation behaviors of the samples were investigated using a dynamic mechanical analyzer (DMA) (Q800, TA Instrument). The dimensions of the DMA samples were 13 mm × 5 mm × 1 mm (length × width × thickness), and the heating rate was 5 °C/min. The stress relaxation test was carried out at 130 °C under a strain of 5%. The dimensional changes of the samples were characterized using a thermomechanical analyzer (TMA) (Q400, TA Instruments) heated at 5 °C/min. Atomic force microscopy (AFM) (Multimode-8, Bruker, Billerica, MA, USA) was employed to observe the micro-phase separated structures of S-PU and H-PU. The changes in the morphological features of the self-healed PU films were observed by field emission scanning electron microscopy (FE-SEM) (AIS2100, SERON, Uiwang-si, Korea). The tensile properties and self-healing efficiencies of the prepared PU samples were investigated employing a universal testing machine (UTM) (LR5K Plus, LLOYD, West Sussex, UK). All measurements in the tensile tests were carried out at 25 °C with a cross-head speed of 500 mm/min. The oscillatory shear tests were conducted employing a rheometer (TA, AR2000, New Castle, DE, USA).

The samples were placed on the 25 mm parallel plate of the rheometer at room temperature and heated to 210 °C. The samples were retained at 210 °C with N_2_ gas purging until they had fully melted. The gap was set to 1.0 mm. The frequency sweep (from 620 to 0.01 rad/s) were then performed under 0.1% strain after trimming.

## 3. Results and Discussion

### 3.1. Synthesis of S-PU and H-PU

**Figure 1:**
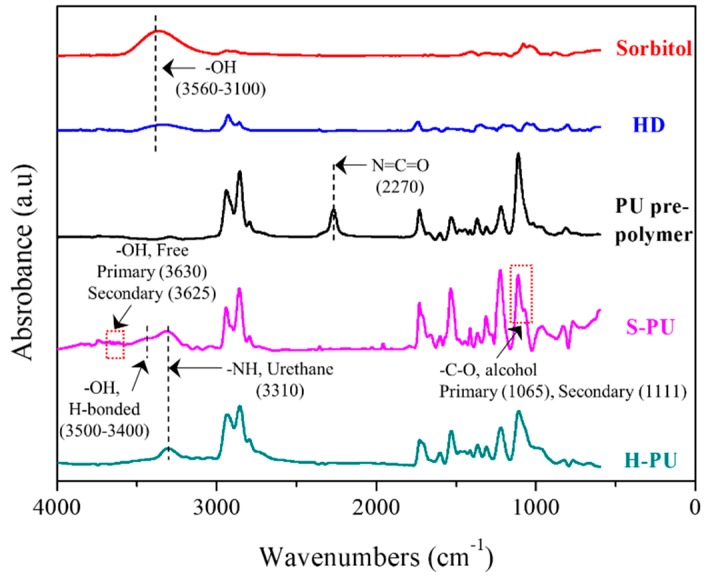
FT-IR spectra of the chain extenders (sorbitol and HD), PU prepolymer, S-PU, and H-PU.

The chemical structures of S-PU and H-PU were characterized by their FT-IR spectra shown in Figure 1. The peak at 2270 cm^−1^ corresponding to the N=C=O groups of PU prepolymers did vanish after the chain extension reaction with sorbitol or HD. In the spectrum of chain-extended S-PU, the peak due to –NH stretching at 3310 cm^−1^ is attributed to urethane linkage formation [48]. The hydrogen-bonded (H-bond) –OH stretching at 3500–3400 cm^−1^ and the free –OH stretching at 3630–3625 cm^−1^ are attributed to the free –OH groups of sorbitol, which did not participate in the urethane linkage formation. Although the primary –OH of sorbitol is more reactive with N=C=O than the secondary –OH [45,46,47], the spectrum exhibits the characteristic –C–O peaks of both the secondary and the primary hydroxyl groups at 1111 and 1065 cm^−1^, respectively (Supplementary Material, Figure S1). This indicates that during the chain extension, some of the secondary –OH groups reacted with the N=C=O group of the PU prepolymer. On the other hand, the H-PU spectrum (with no free –OHs) exhibits only the –NH stretching of the urethane unit (3310 cm^−1^) and the –C–O–C– stretching of polyol (1107 cm^−1^) (Supplementary Material, Figure S1) [49,50,51,52]. The molecular weights (M_n_ and M_w_) and PDIs measured with GPC for S-PU and H-PU are summarized in Table 1 (Raw data of GPC in Supplementary Material, Figure S2). The molecular weights and PDIs of S-PU and H-PU are similar because both polymers were prepared by the same isocyanate–hydroxyl group reaction between the PU prepolymer and the hydroxyl chain extender (sorbitol or HD).

**Figure 2:**
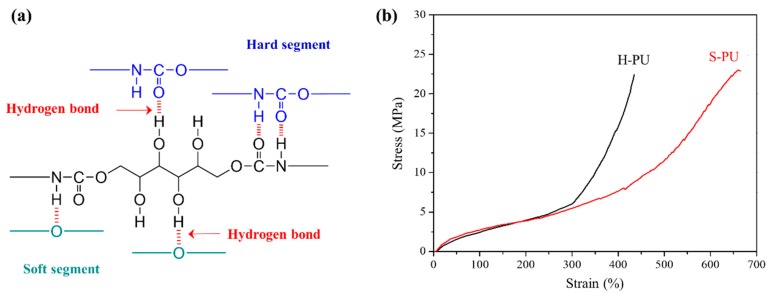
Characteristics of S-PU investigated: (**a**) possible hydrogen bonds in S-PU; (**b**) tensile properties of S-PU and H-PU at 25 °C.

Figure 2a shows the possible H-bonds in S-PU. In conventional polyether–polyol-based PU systems, the –NH and C=O units of urethane inter- or intra-molecularly interact with the –C–O–C units of polyol, confirming that H-bonding of the urethane moiety is possible [53]. However, the free –OH groups of S-PU can form further H-bonds in the hard and soft domains, as illustrated in Figure 2a. The formation of H-bonding with unreacted –OH of S-PU was investigated in the FT-IR spectra (see Figure S1). The strength of H-bonds largely affected the thermal and mechanical properties of S-PU. In conventional PUs prepared from the PU prepolymer and diols, a micro-phase-separated structure resulting from the partial solubility of the hard and soft domains is usually seen [54,55]. The DSC thermogram of H-PU given in Figure S3 revealed clear glass transition temperatures (T_g_) of the hard and soft segments. Meanwhile, as the temperature increased in the DMA measurements in Figure S4, a typical glass-rubbery transition with rubbery plateau region appeared, reflecting the well-organized micro-phase separated structures. On the other hand, the glass transition temperature of the soft segment was not seen in the DSC thermogram of S-PU (Figure S3), and the low association in the hard domains caused a rapid decrease in the storage modulus in the rubbery plateau region of the DMA data, with an increased loss factor (tan δ) (Figure S4) [54,55]. Figure 2b shows the stress–strain curves of S-PU and H-PU determined in tensile tests. The mechanical properties (elongation at break (ε) and stress value (σ)) were higher in S-PU than in H-PU. Specifically, the values of ε and σ were 658% and 22.9 MPa, respectively, in S-PU, versus 434% and 20.5 MPa, respectively, in H-PU. The higher mechanical properties of S-PU are attributed to the formation of H-bonds as displayed in Figure 2a. Although the H-bonding improved the σ and ε of S-PU, the strain hardening effects were slightly delayed, occurring around 430% strain in S-PU, versus 300% strain in H-PU. The strain hardening of H-PU at lower strain is attributable to the micro-phase separated structure of the hard and soft domains [56]. On the other hand, the relatively less micro-phase-separated structure of S-PU, caused by the H-bond formation, weakened the strain hardening in tensile test. The difference in micro-phase separation of S-PU and H-PU could be confirmed in AFM images given in Figure S5. H-PU showed domains of hard segments in submicron size while S-PU did not due to the relatively less micro-phase separated structures. It is speculated that the hydroxy urethane unit introduced for the exchange reaction with the urethane chains lowered the micro-phase separation of S-PU over the control PU (H-PU), but the hydrogen bonds formed by the hydroxyl groups conferred excellent mechanical properties, comparable to those of H-PU.

### 3.2. Exchange Reaction and Thermomechanical Properties of S-PU

**Figure 3:**
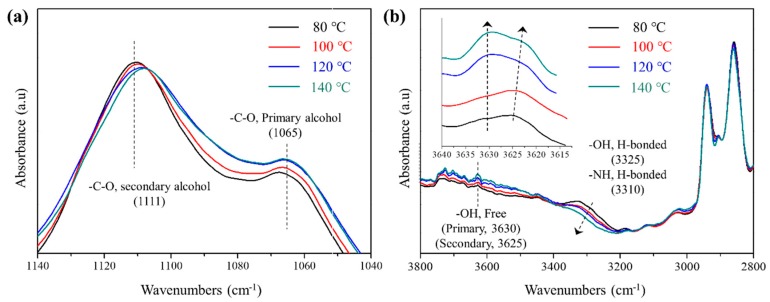
Temperature-dependent FT-IR spectra of S-PU: (**a**) at low wavenumbers (1140–1040 cm^−1^); (**b**) at high wavenumbers (3800–2800 cm^−1^).

**Figure 4:**
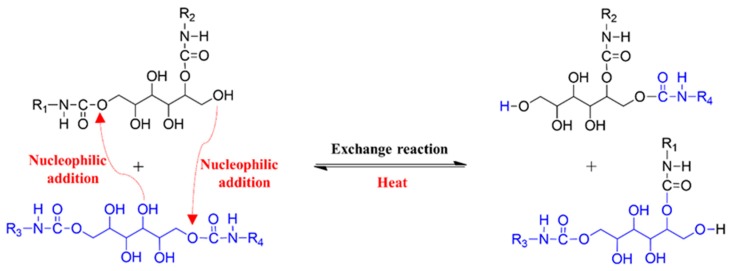
Schematic of the exchange reaction among the hydroxy urethane units of S-PU.

Exchange reactions of carbamates initiated by free –OH groups are the probable exchange reaction among the hydroxy urethane units at elevated temperatures. Exchange reactions occur via nucleophilic addition of –OHs to carbamate under thermal activation and are temperature-dependent [29,32]. To investigate the exchange reaction mechanism among the hydroxy urethane units of S-PU, the FT-IR peaks corresponding to the free hydroxyl groups of S-PU were monitored during the heating process (from 80 to 140 °C) and shown in Figure 3 after the normalization using peaks of methylene units. The peak intensities of –C–O at 1065 cm^−1^ corresponding to the primary hydroxyl groups were gradually increased with increase of temperature by the exchange reaction given in Figure 4. In contrast, the –C–O peaks at 1111 cm^−1^ corresponding to the secondary –OH of sorbitol were reduced a little and shifted to lower wavenumbers with increasing temperature. Peaks due to free –OH groups at 3625 and 3630 cm^−1^ are attributable to secondary –OH and primary –OH groups, respectively. It was observed that the peak intensities at 3630 cm^−1^ increased while peak at 3625 cm^−1^ decreased with increase of the temperature. These spectroscopic features are attributed to the thermally activated exchange reaction among the –OH and carbamate groups of S-PU molecules as described in Figure 4 [29,32]. However, in the H-PU spectrum, increasing the temperature altered the peak intensities and shifted the –NH (H-bonded urethane) and –C–O–C (ether in polyol) peaks toward lower wavenumbers (Supplementary Material, Figure S6). These trends correspond to the dissociation of H-bonds in H-PU. The exchange reaction occurring in S-PU is absent in H-PU because the hydroxyl group enabling the uncatalyzed exchange reaction of carbamate groups is lacking. According to these results, the hydroxyl unit is essential for the efficient exchange reaction among the hydroxyl-urethane units under mild conditions (relatively low temperature at ambient pressure without catalyst).

**Figure 5:**
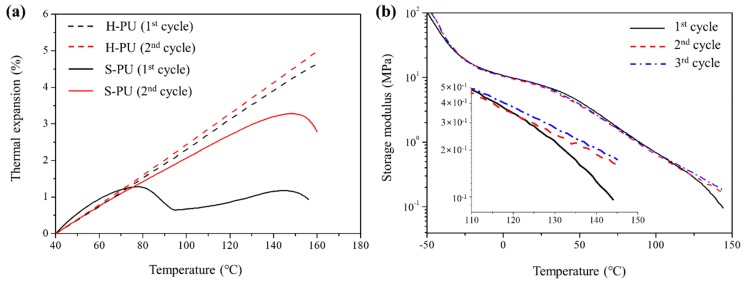
Thermal properties of S-PU and H-PU during the repeated heating cycle: (**a**) thermal expansion measured by TMA; (**b**) storage modulus versus temperature of S-PU in repeated DMA measurements.

The thermally activated exchange reaction of S-PU was probed by TMA. Figure 5a shows the dimensional changes in S-PU and H-PU measured by TMA. The coefficients of thermal expansion (CTEs) are summarized in Table 2. During the first and the second cycle, H-PU shows the typical linear expansion of PU [57,58], with no significant change in the CTE. However, the TMA of S-PU yielded two maximum points, at 79 °C and 149 °C. In the initial state, S-PU exhibited similar thermal expansion to H-PU. However, the dimensions of S-PU decreased after 79 °C, possibly because the thermally activated exchange reaction at 79 °C altered the configuration of this polymer (see Figure 4). The dimensional reduction was followed by a gradual increase in thermal expansion, although the CTE was significantly lower in the high temperature range than in the low temperature range (40~79 °C), as shown in Table 2. Above 149 °C, the CTE was reduced by the exchange reaction accompanying the stress relaxation [59]. On the other hand, the CTE of H-PU was much higher than that of S-PU above 79 °C and did not decrease with further heating. These results reveal that the exchange reaction of S-PU molecules restricts the thermal expansion of the polymer matrix. Unlike the first cycle, the second measurement yielded no dimensional reduction at 79 °C and the thermal expansion was still suppressed by the exchange reaction (as evidenced by the reduced CTE). Furthermore, the maximum temperature of TMA thermogram in S-PU increased from 149 to 152 °C, implying that S-PU was configurationally rearranged by the exchange reaction. The effect of configuration change in S-PU was also confirmed by the repeated heating and cooling tests in DMA. Although the DMA thermograms of H-PU remained constant, the lowered micro-phase separation caused by three repeated tests of heating and quenching reduced the storage modulus, *G*′, of S-PU (Supplementary Material, Figure S7). Lowered micro-phase separation also slightly reduced the G′ in the rubbery plateau region of S-PU (Figure 5b). However, at higher temperatures (above 125 °C), the *G*′ of S-PU clearly improved in cycles 2 and 3 (Figure 5b, inset), implying that the exchange reaction affected the configuration change in S-PU, conferring a partial gel-like property.

**Figure 6:**
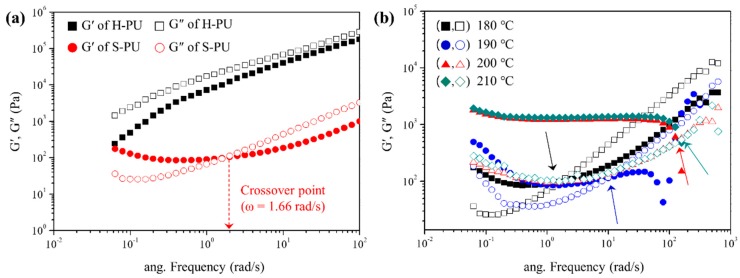
Rheological properties of S-PU and H-PU: (**a**) Storage moduli (*G*′, filled symbols) and loss moduli (*G*″, open symbols) versus angular frequency of S-PU (red) and H-PU (black) at 180 °C; (**b**) G′ and G″ versus angular frequency of S-PU at 180 °C, 190 °C, 200 °C and 210 °C.

To clarify how the exchange reaction affects the rheological properties of the polymer systems, we investigated the dynamic rheological properties of S-PU and H-PU under oscillation mode at 180, 190, 200, and 210 °C. At elevated temperatures, S-PU showed good thermal stability with no dissociation of the urethane unit or by-product formation through side reactions (Supplementary Material, Figure S8). Owing to its linear structure, H-PU exhibited liquid-like behavior (i.e., G′ < G″) over the whole range of available angular frequencies at 180 °C as shown in Figure 6a. S-PU exhibited similar liquid-like behavior at high frequencies (G′ < G″), but its flow resistance dramatically increased with decreasing angular frequency. After the crossover point (where G′ = G″) at 1.62 rad/s, S-PU exhibited a solid-like property (G′ > G″), which implies the formation of a cross-linked gel-like structure [60,61,62,63]. This transition of S-PU from G′ < G″ to G′ > G″ at low frequencies was attributed to the exchange reaction accompanying the formation of gels. Low frequencies provide a sufficient time scale for active exchange with abundant –OH groups. The resultant gel formation by the exchange reaction was also evidenced by the increased complex viscosity (η*) after the crossover point (Supplementary Material, Figure S9) [63,64].

Figure 6b gives the frequency dependencies of G′ and G″ in S-PU at different temperatures, and Table 3 lists the crossover points at each temperature. As the temperature increased, the rate of the exchange reaction increased, so the crossover points were found at higher frequencies (shorter time scales). G′ also increased with temperature, implying that the rate of gel formation was controlled by the rate of the exchange reaction. On the other hand, in H-PU (which lacks the hydroxy unit), the exchange reaction was not activated and the G′-to-G″ crossover was not observed until 210 °C. This implies a sluggish gel formation by the exchange reaction among the carbamate groups, which was activated at high temperatures by –OH groups (Supplementary Material, Figure S10) [27,28,29,30]. That is, the hydroxy urethane unit of S-PU enables the exchange reaction between –OH and carbamate groups at lower temperatures than the exchange reaction among carbonate groups in H-PU. The hydroxy urethane unit is promising for realizing a self-healing polymer, as it initiates the exchange reaction at relatively low temperature, thereby minimizing damage by oxidation or side reactions.

### 3.3. Self-Healing

**Figure 7:**
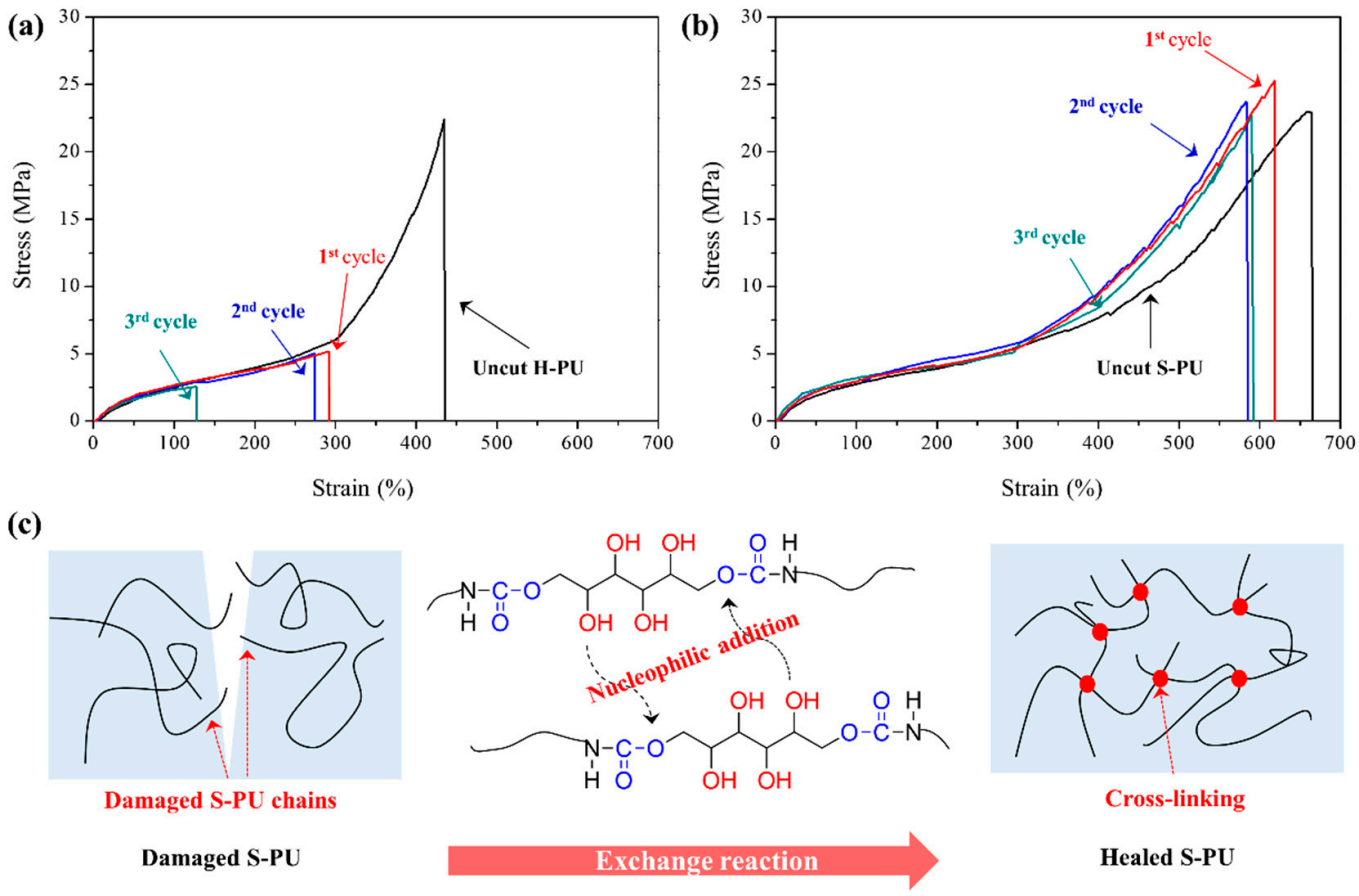
Results of self-healing at 130 °C for 120 min: (**a**) tensile properties of H-PU after self-healing; (**b**) tensile properties of S-PU after self-healing; (**c**) schematic of the self-healing mechanism and configurational change of typical S-PU after the exchange reaction.

The self-healing properties of H-PU and S-PU were investigated in a tensile test of pristine (uncut) and healed samples after cut. For the self-healing tests, the dog-bone shaped H-PU and S-PU samples were cut into two pieces by a razor blade. The pieces were then contacted closely and incubated for 120 min in a convection oven at 130 °C (Supplementary Material, Figure S11). Figure 7a,b shows the tensile test results of the uncut and self-healed samples of H-PU and S-PU, respectively. To evaluate the ability of self-healing of S-PU and H-PU, repeated cutting-healing tests with specimens for tensile tests were performed up to three times on same samples and the results of the tensile tests are summarized in Table 4. After self-healing, the values of σ, ε and E of H-PU were largely reduced when cutting-healing test was repeated up to three times because H-PU self-heals by a physical mechanism based on the diffusion of molecules above T_g_ [65,66]. As already confirmed by the rheological properties, the carbamate groups spontaneously exchange at high temperatures (about 210 °C) without catalysts. On the other hand, the tensile properties of S-PU maintained its integrity after repeated cutting-healing tests, because physical self-healing was combined with the exchange reaction activated by the nucleophilic addition of free –OH groups to carbamates, which occurred at a lower temperature than the carbamate exchanges in H-PU.

In the tensile test, the tensile strength (σ) and Young’s modulus (E) of S-PU were found to be slightly increased after self-healing and post curing process (Figure S12 and Table S1). This improvement may be attributed to the cross-linked gel-structure formed by the thermally activated exchange reaction, as illustrated in Figure 7c. The increased gel fraction in healed S-PU was also confirmed by gel fraction measurements in DMF. The gel fraction of S-PU almost doubled after the thermal treatment of the S-PU sample for self-healing or post cure (Table 4 and Table S1). In other words, the exchange reaction in S-PU not only induces recombination among the chains of the damaged S-PU, but also improves the mechanical properties through the configuration change from linear to gel-like. The self-healing efficiencies of S-PU and H-PU given in Table 4 were obtained by the following equation,

(1) $$\mathrm{Self}–\mathrm{healing}\;\mathrm{efficiency}=(\sigma_{\mathrm{healed}}/\sigma_{\mathrm{post}-\mathrm{cured}})\times 100\;\%$$

where σ_healed_ and σ_post-cured_ are tensile strengths of self-healed and undamaged post cured samples, respectively. In Table 4, the self-healing efficiencies of S-PU were excellent while those of H-PU were relatively low and decreased very much in repeated tests. The difference in self-healing ability between S-PU and H-PU was also investigated in an SEM analysis of the cut damage and self-healed samples. The damage on the S-PU surface was almost fully repaired after heating at 130 °C for 120 min, whereas the damage on H-PU surface was hardly recovered (Supplementary Material, Figures S13 and S14).

## 4. Conclusions

A self-healable sorbitol-based PHU (S-PU) was prepared by simply introducing sorbitol as a bifunctional chain extender of the PU prepolymers. The hydroxy urethane units in S-PU not only improved the tensile properties over conventional PU, but also conferred excellent self-healing properties. The former improvement is attributable to the formation of hydrogen bonds of hydroxyl groups; the latter is conferred by nucleophilic addition of hydroxyl groups to the carbamate units without a catalyst. Moreover, the exchange reaction caused a configuration change from a linear to a cross-linked structure, leading to reversible gel formation with enhanced mechanical properties and suppressed thermal expansion during the heating process. A facile and environmentally friendly material for use in coatings, adhesives, and elastomers could be prepared by the suggested synthetic framework for PHU.

## Figures and Tables

**Table 1:** Composition and molecular weights of the prepared PUs.

Sample	Composition (Molar Ratio)				M_n_ (g/mol)	*M*_w_ (g/mol)	PDI
	MDI	PTMEG	Sorbitol	HD			
S-PU	2	1	1	–	56,600	126,900	2.24
H-PU	2	1	–	1	64,600	131,900	2.04

**Table 2:** The coefficients of thermal expansion (CTEs) of S-PU and H-PU.

	CTE (1/°C)			
	1st Cycle		2nd Cycle	
S-PU	0.484 ^a^	0.132 ^b^	0.398 ^a^	0.306 ^b^
H-PU	0.436		0.459	

^a^ CTE at 40~79 °C. ^b^ CTE at 100~140 °C.

**Table 3:** Angular frequencies of *G*′-to-*G*″ crossover in S-PU and H-PU at different temperatures.

	S-PU				H-PU			
	180 °C	190 °C	200 °C	210 °C	180 °C	190 °C	200 °C	210 °C
ω (rad/s)	1.62	9.55	131.57	159.47	–	–	–	0.08

**Table 4:** Tensile properties and gel fraction of S-PU and H-PU after repeated cutting/self-healing cycles.

Properties	S-PU				H-PU			
	Uncut S-PU	Self-Healed S-PU			Uncut H-PU	Self-Healed H-PU		
		1st	2nd	3rd		1st	2nd	3rd
σ (MPa)	22.92	25.24	23.76	22.67	22.53	5.182	4.541	2.554
ε (%)	660	620	580	590	430	290	260	130
E (MPa)	4.447	4.893	5.012	5.221	4.655	4.353	4.117	3.832
Self-healing efficiency (%)	–	93.79	88.29	84.24	–	22.68	19.87	11.17
Gel-fraction ^a^ (%)	25.4	52.7	53.2	53.1	35.8	36.2	36.3	35.2

^a^ The gel fractions were calculated as (*W*_1_ − *W*_2_)/*W*_1_ × 100 (where *W*_1_ is the initial sample weight and *W*_2_ is the weight of the residual solid contents after immersing the sample in DMF for 5 h at 25 °C).

## Supplementary Materials

The supplementary materials are available online.
