# Supplementary material for: Sorbitol as a Chain Extender of Polyurethane Prepolymers to Prepare Self-Healable and Robust Polyhydroxyurethane Elastomers
Source: Molecules. 2018 Sep 30;23(10):2515. doi: 10.3390/molecules23102515 (PMC6222304; doi:10.3390/molecules23102515)
Supplement: Supplementary file 1 [file molecules-23-02515-s001.pdf]

Supplementary Material

Sorbitol as a chain extender of polyurethane prepolymers to prepare self-healable and robust polyhydroxyurethane elastomers

Sang-Hyub Lee, Se-Ra Shin and Dai-Soo Lee*

Department of Semiconductor and Chemical Engineering, Chonbuk National University, 567 Baekje-daero, Deokjini-gu, Jeonju-si, Chonbuk, 54896, Republic of Korea

* Correspondence: daisoolee@jbnu.ac.kr; Tel.: +82-63-270-2431

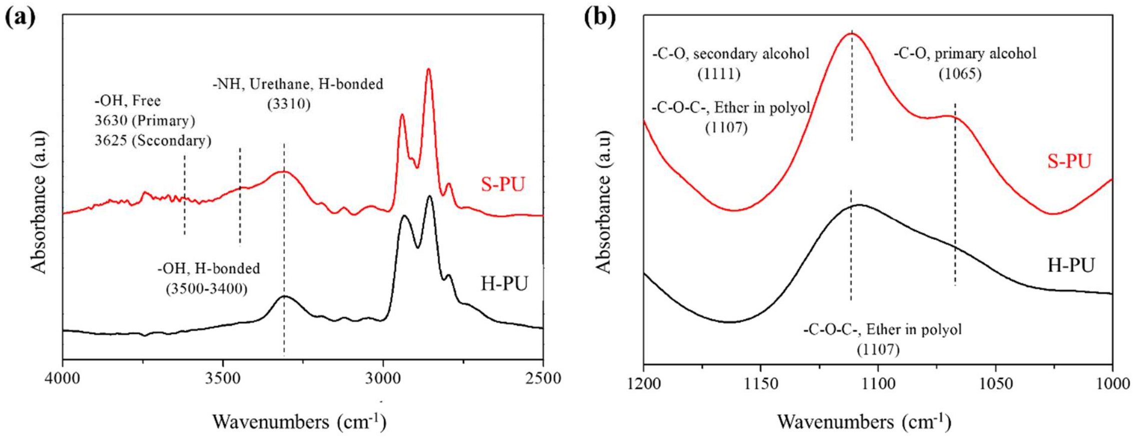

Figure S1. Enlarged views of the FT-IR spectra of S-PU and H-PU: (a) from 4000 to 2500 cm^{-1}; (b) from 1200 to 1000 cm^{-1}.

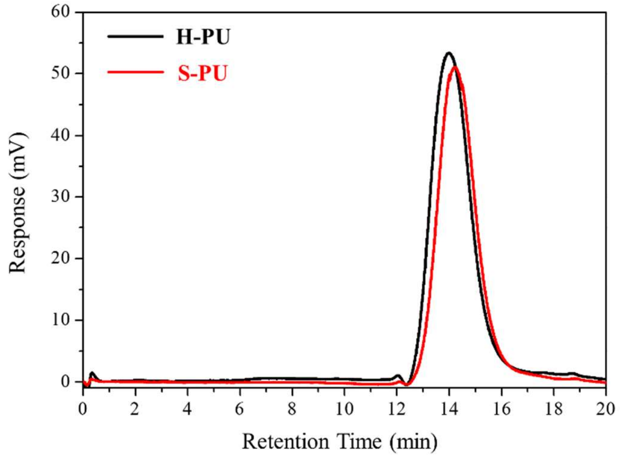

Figure S2. GPC traces of the synthesized S-PU and H-PU.

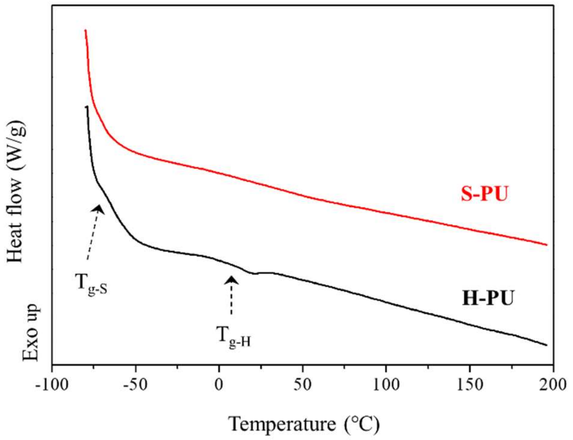

Figure S3. DSC thermogram of S-PU and H-PU.

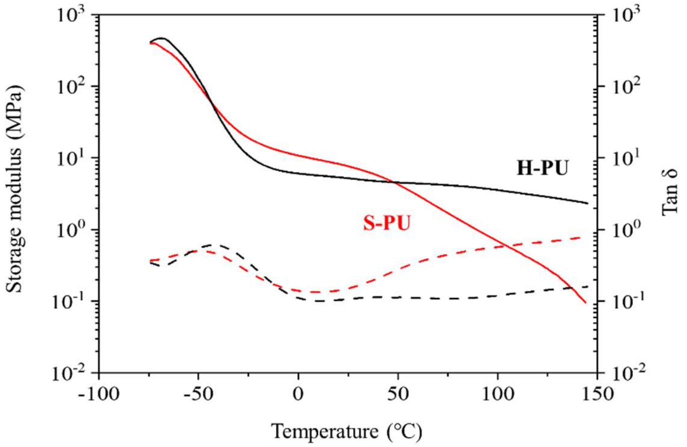

Figure S4. Storage moduli (solid lines) and tan δ values (dotted lines) of S-PU (red) and H-PU (black) obtained in the DMA measurements.

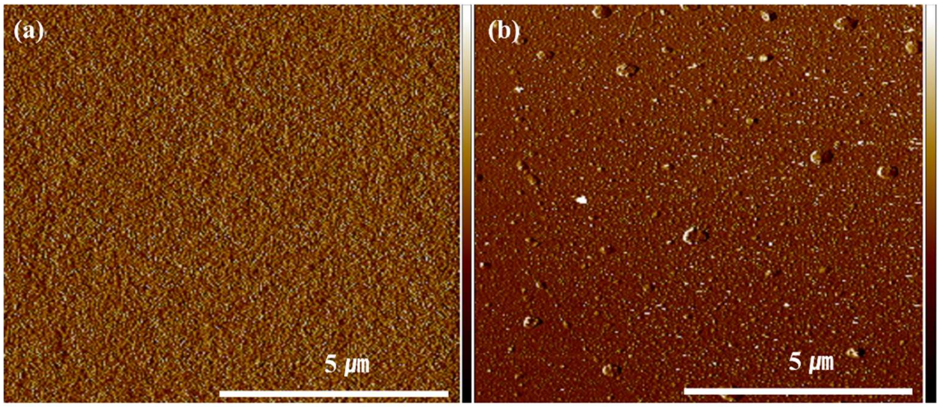

Figure S5. AFM images in the phase mode: (a) S-PU; (b) H-PU.

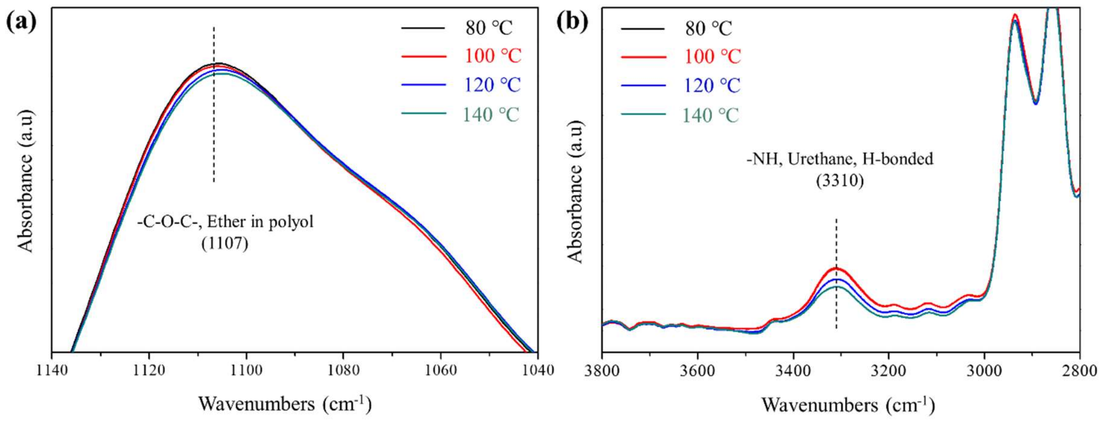

Figure S6. Temperature dependent FT-IR spectra of H-PU: (a) Absorbance at low wavenumbers (1140–1040 cm^{-1}); (b) Absorbance at high wavenumbers (3800–2800 cm^{-1}).

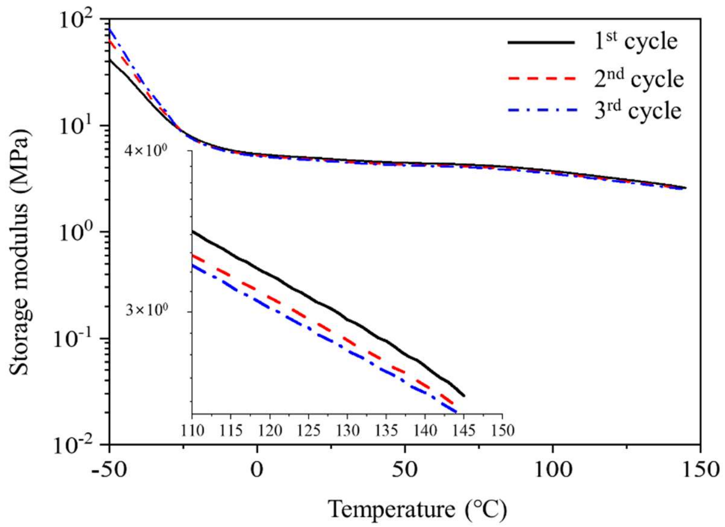

Figure S7. Storage modulus versus temperature curves of H-PU in repeated DMA measurements.

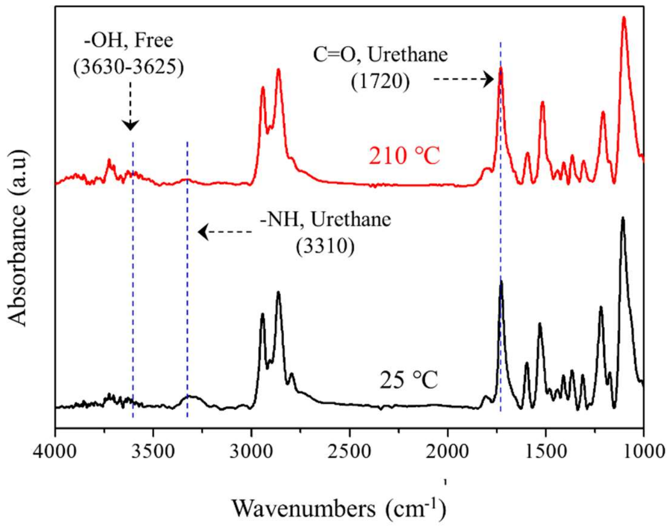

Figure S8. Normalized FT-IR spectra of S-PU at 25 °C (black) and 210 °C (red).

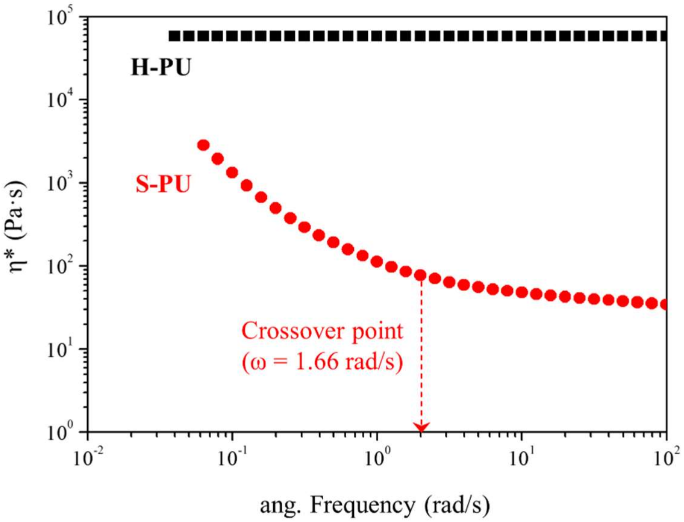

Figure S9. Complex viscosity versus angular frequency of S-PU and H-PU at 180 °C.

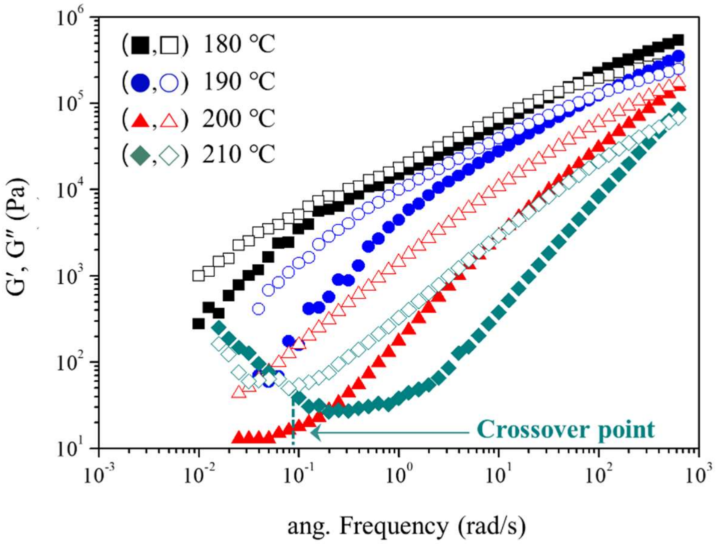

Figure S10. *G*′ (filled symbols) and *G*″(open symbols) of H-PU versus angular frequency at 180 °C, 190 °C, 200 °C and 210 °C.

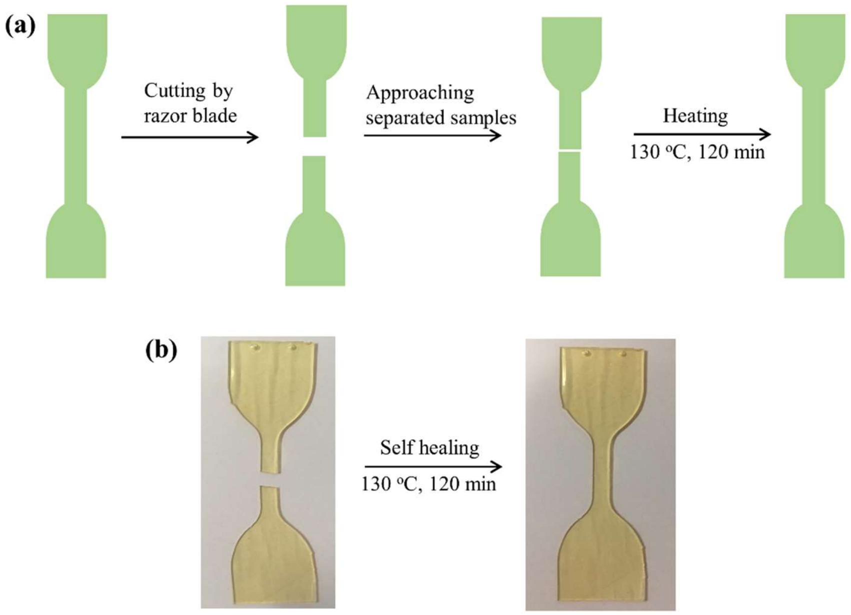

Figure S11. Preparation of specimens for self-healing tests by UTM: (a) schematic for the preparation of the sample specimens; (b) digital camera images of the S-PU sample after cutting (left) and self-healing at 130 °C for 120 min.

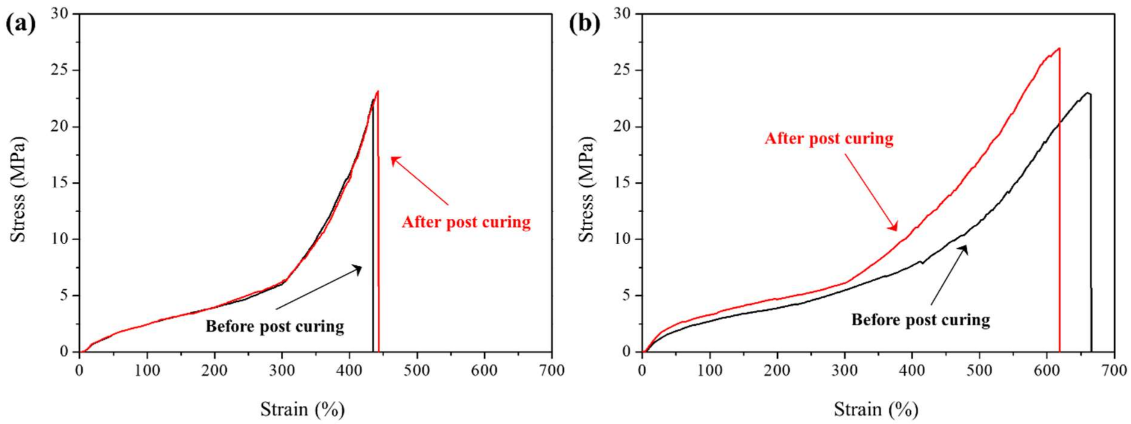

Figure S12. Effects of post curing at 130 °C for 120 min: (a) tensile properties of H-PU before (black) and after (red) post curing; (b) tensile properties of S-PU before (black) and after (red) post curing.

Table S1. Tensile properties and gel fraction of S-PU and H-PU after post curing at 130 °C for 120 min

	S-PU		H-PU	
	Uncut S-PU	Post cured S-PU	Uncut H-PU	Post cured H-PU
σ (MPa)	22.92	26.91	22.53	22.85
ε (%)	658.3	619.5	433.8	434.5
E (MPa)	4.447	5.952	4.655	4.641
Gel- fraction [a] (%)	25.42	54.94	35.78	35.42

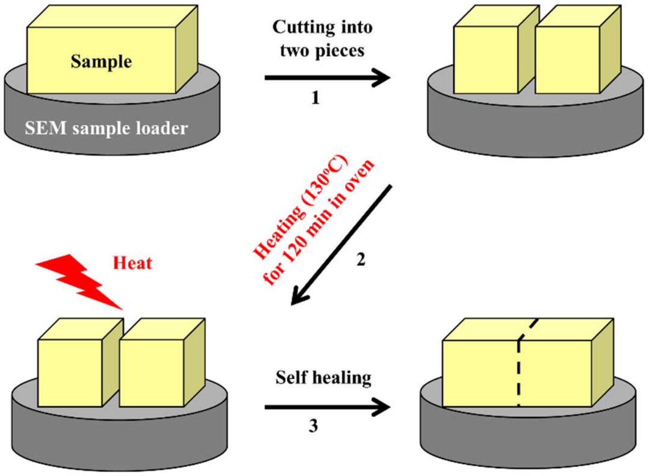

Figure S13. Schematic of the sample preparation to observe the self-healing effects by SEM.

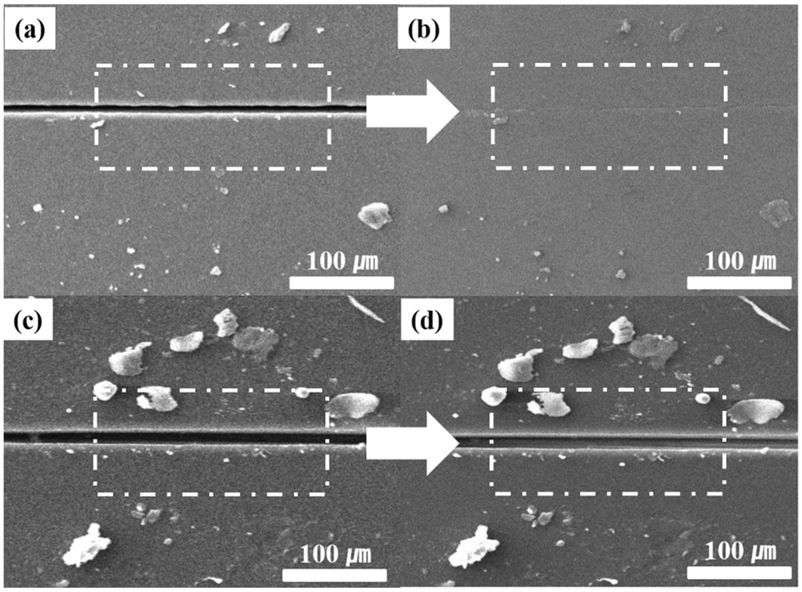

Figure S14. SEM images of the PUs after cutting and heating to 130 °C for 120 min: (a) cut S-PU; (b) self-healed S-PU after heating; (c) cut H-PU; (d) unhealed H-PU after heating.
